# Critics of Present Conditions: Dawn of a Brighter Era: College Plans

**Published:** 1919-05-03

**Authors:** 


### FUTURE OF NURSING DISCUSSED.

### Critics of Present Conditions: Dawn of a Brighter Era: College Plans.

A PEACE Nursing and Midwifery Conference was held this week in connection with the Nursing Exhibition in London, and in the Mortimer Hall, Mortimer Street, and St. Andrew's Hall, Newman Street, interesting and instructive papers were read before audiences which in some instances were gratifyingly large.

### A CRITIC OF THE NURSING SYSTEM.

"Should Nursing be a Profession?" was the title of a paper by Mr. H. C. Crouch, M.R.C.S., L.R.C.P. The tone of the address was quietly sarcastic throughout at the expense of employers of nurses. Before reading his paper Dr. Crouch gave a warning that he was going to tread on people's corns. Nurses, he said, had always, since their religious origin, been exploited by the public, and it was time a radical alteration took place. He hoped that soon such a thing as a Flag Day for the assistance of nurses would be unthinkable. Hospitals could not be run without nurses any more than without subscribers, but he had never yet read a hospital report in which those who had given up their health had been thanked so warmly as those who had given their money. Nurses were overworked and underpaid. He had seen probationer after probationer break down as the result of the strain of their training, and it was a tragedy that at forty years of age a nurse should find that she was no longer in demand. Under present conditions of hours and work it was impossible for a young nurse to study properly, or even to thoroughly understand what she is doing when on duty.

### REFORMS TO COME.

He hoped the College of Nursing would bring about reforms. In fact, he could not understand why it had not already done so, seeing that the Council consisted mainly of people in the highest authority in hospitals, who had the power in their hands. Nurses, he maintained, should not suffer for the benefit of patients; that was a wrong principle. There was no justification for the sacrifice of a single woman in the interest of any patient in hospital, and it was not necessary. If nursing was to be a profession nursing students should give their services in return for the training they received, just as the students in other professions did, and the doles of £10 or £15 a year should stop. But when nurses had finished their training they should be in a position to demand proper remuneration, and not the miserable salaries offered to them now. There was a scarcity of candidates for nursing. The "nurses of to-morrow" evidently had not the slightest intention of joining hospital staffs under present conditions, and the sooner that was recognised the better. Dr. Crouch had much criticism to offer on the present methods of training in hospitals, but acknowledged that the sister-tutor plan of the College of Nursing was a step in the right direction. As to the higher-curriculum scheme, which he understood a committee of matrons was going to draw up, he predicted it would be a failure. Matrons had not the wide outlook necessary. Let the education authorities take this matter in hand.

### NURSES MUST HELP THEMSELVES.

Miss A. C. GIBSON (former matron of Birmingham Infirmary) dealt with "State Registration: its Effect on the Nursing Profession." The lack of uniformity in the training of nurses was, she urged, a distressing feature of present conditions. There should be a fixed standard, and State Registration—to which she was a recent convert—would do much to bring that about. Nurses holding certificates from first-class training-schools were handicapped by the competition of nurses from less efficient establishments, but State Registration, by insisting upon a standard training, would alter that. The time had come when nurses must fight their own battles. It was fashionable to talk as though matrons wished to hold all power in their own hands, but she had never yet met a matron who did not desire that her nurses should have the opportunity to act in their own interests. But it was a pathetic fact that nurses would not help themselves. They must shed this indifference, and also get rid of their prejudices and cliques. The post-graduate courses designed by the College of Nursing would enable nurses to take up any special branch desired; at present she admitted they were too hard worked as a rule to go in for specialisation.

### NURSES ON THE COLLEGE COUNCIL.

Miss SPARSHOTT (matron, Royal Infirmary, Manchester) explained to a large audience what the College of Nursing had done, and what it hopes to do. The fact that the College membership was 13,000 and still growing showed its popularity and its need. Its register was now in print, and would soon be published; it would cost £1,000. The elections for the Council would shortly be taking place, and nurses should take a real interest in them. As to the future, the College intended to go forward and help nurses to a higher standard of education, higher wages, and, she hoped, shorter hours, and in carrying out this programme it was a great advantage that the College was recognised by all the training-schools. If nurses were not satisfied with members of the Council, get rid of them; the College wanted nurses themselves to take responsibility. "Hitherto," she proceeded, "nurses have been accustomed to matrons doing the work for them, but we should only be too glad if nurses would come forward and help us in the work of the Council." At present, she admitted, it was difficult for nurses to get on the Council, because as a rule they were not widely known; but that difficulty must be overcome.

### MENTAL NURSES' ASPIRATION.

Mr. J. F. POWELL, M.R.C.S., Hon. Secretary, Asylum Workers, Association, spoke on "Mental Nursing: the Effect of State Registration," and pointed out the value of a double qualification. At the present time, to obtain the higher posts in mental hospitals it was, he said, almost a *sine qua non* that a candidate should have had training in mental diseases, and also a general hospital training. A large number of hospital nurses were entering mental hospitals to qualify for these posts, and he urged that it would be a good thing if there could be reciprocity, whereby training in a mental hospital would count as part of the probationary period in a general hospital. A State Register of Nurses would, he thought, act as an incentive to nurses in mental hospitals, especially among the newcomers, who would not rest satisfied until they had reached the highest status. When the Register is formed he hoped the mental nurse would become recognised as an integral part of the nursing profession.

**Future of Nursing Discussed**—(*continued*).

### The Care of Mothers and Babies.

"The Co-ordination of Infant-Welfare Work and Its Development" was a subject dealt with in an interesting way by Miss Halford (Secretary National League for Health, Maternity and Child-Welfare). There could, she said, be no thoroughly satisfactory infant-welfare work until the co-ordination of local authorities had been secured. At present too many local authorities had their fingers in this particular pie. When these authorities had been co-ordinated all who were working for infant-welfare should join them; and the great aim should be to help the mother—not to make statistics. Local authorities, she complained, were cheeseparing and would not employ sufficient workers to accomplish the task efficiently. For one thing co-ordination would prevent the mother being "over-visited" in her home, a thing which she disliked. Nurses had a great future before them in this branch, for, as a nation, we should in the future have to deal more effectively with minor ailments. But nurses must realise that it was not so much a question of cure as of prevention. If the new ante-natal clinics were to be a success the co-operation of midwives must be secured; and, further, nothing could be done to encourage breast-feeding on right lines unless midwives came to the rescue. Voluntary workers, too, were necessary. She realised that was a thorny subject, but appealed to nurses to cast aside their prejudices. Closer co-operation with the doctor would also be called for. Further, they must bring the dentist to the mother, and get rid of the sometimes terrible state of things, which was poisoning, not only the mothers, but their babies too. More institutional treatment was necessary; and she looked forward to the time when there would be convalescent homes for mothers and their babies. In a discussion which followed, criticism was levelled against the treatment accorded expectant mothers at certain ante-natal clinics. Dr. E. Pritchard, who was in the chair, agreed that there might be ground for complaint, but pleaded that the clinics were only in their infancy, and abuses, if any, would be rectified in the light of experience.

